# Tubular and lamellar hydrogen-bonding molecular assemblies of isophthalic acid derivatives bearing a –CONHC_*n*_H_2*n*+1_ chain†

**DOI:** 10.1039/c8ra04077j

**Published:** 2018-06-19

**Authors:** Chao Lv, Takashi Takeda, Norihisa Hoshino, Tomoyuki Akutagawa

**Affiliations:** Graduate School of Engineering, Tohoku University Sendai 980-8579 Japan; Institute of Multidisciplinary Research for Advanced Materials (IMRAM), Tohoku University 2-1-1 Katahira, Aoba-ku Sendai 980-8577 Japan akutagawa@tohoku.ac.jp +81-22-217-5653; Institute of Materials, China Academy of Engineering Physics Jiangyou 621908 Sichuan P. R. China

## Abstract

Isophthalic acid derivatives (CnIP), bearing alkylamide chains (–CONHC_*n*_H_2*n*+1_: *n* = 6, 10, 14, and 18) at the 5-position that can participate in hydrogen bonding, were prepared and evaluated for their hydrogen-bonding molecular assembly structures for organogelation and liquid crystal formation. The hydrogen-bonding carboxylic acid (–COOH) groups form a ring-shaped (CnIP)_6_ hexamer or a one-dimensional (1D) zig-zag (CnIP)_∞_ chain. Although neither organogelation nor liquid crystal formation was observed in the isophthalic acid derivative bearing an alkoxy (–OC_14_H_29_) chain, C14IP and C18IP derivatives could form both organogel and liquid crystal states through intermolecular N–H⋯O = amide-type hydrogen-bonding interactions. A discotic hexagonal columnar liquid crystal (Col_h_) phase was observed in hydrated (C14IP)_6_·(H_2_O)_*n*_ and (C18IP)_6_·(H_2_O)_*n*_, whereas a lamella-type liquid crystal (L_a_) phase was confirmed in the unhydrated C18IP. In the Col_h_ phase, O–H⋯O hydrogen-bonding ring-shaped (C14IP)_6_ and (C18IP)_6_ hexamers assembled to form the tubular molecular assembly stabilized by intermolecular–N–H⋯O = hydrogen-bonding interactions along the tube growth direction, where H_2_O molecules were contained within the hydrophilic space. On the other hand, the N–H⋯O = hydrogen-bonding interactions between the 1D zig-zag (CnIP)_∞_ chains formed a layer-type molecular assembly of the L_a_-phase in the absence of water molecules.

## Abstract 2

Isophthalic acid derivatives (CnIP), bearing alkylamide chains at the 5-position that can participate in hydrogen bonding, were prepared and evaluated for their hydrogen-bonding molecular assembly structures for organogelation and liquid crystal formation.

## Graphical abstract

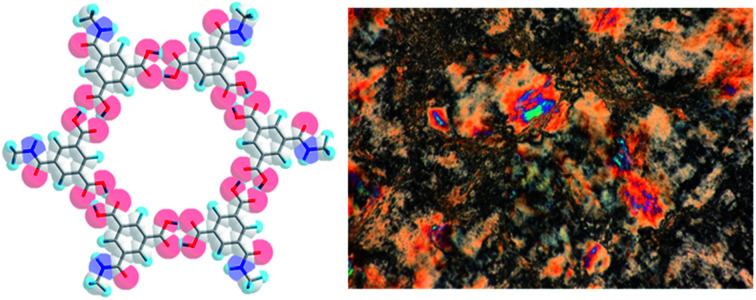

## Introduction

Amide-type N–H⋯O = hydrogen-bonding in polypeptide chains plays an important role in forming the secondary protein structures of α-helices and β-sheets, which are further assembled to form ternary protein structures through weak van der Waals interactions.^1^ The bonding energy of the amide-type hydrogen-binding interaction is approximately 10 kJ mol^−1^, a magnitude favourable for achieving association/dissociation of secondary protein structures at approximately room temperature (*k*_B_*T* ≈ 2.5 kJ mol^−1^).^2–5^ Therefore, amide-type N–H⋯O = hydrogen-bonding interaction is a key intermolecular interaction that controls hierarchical molecular assembly structures. In the field of supramolecular chemistry, directional hydrogen bonding has been used for the construction of various anisotropic molecular assemblies from one-dimensional (1D) chains of phthalic or isophthalic acids,^6–8^ two-dimensional (2D) layers of squaric or trimesic acids,^9,10^ and three-dimensional (3D) diamond-like networks of tetracarboxylic acid derivatives.^11,12^ Among a variety of directional hydrogen-bonding interactions such as O–H⋯O, N–H⋯O, and N–H⋯N, amide-type N–H⋯O = hydrogen-bonding has been used to design single crystal organic ferroelectrics^13–17^ and discotic hexagonal columnar liquid crystal (Col_h_) phases of benzene derivatives bearing multiple alkylamide (–CONHC_*n*_H_2*n*+1_) chains.^18–22^ For instance, ferroelectric polarization (*P*)–electric field (*E*) hysteresis curves have been observed for *N*,*N*′,*N*′′-trialkyl-1,3,5-benzenetricarboxamide (3BC) derivatives; a response is achieved by collective inversion of the 1D N–H⋯O = hydrogen-bonding interactions along the π-stacking column.^18–22^ The application of the opposite *E*-value along the hydrogen-bonding column inverts the direction of the macro dipole moment arising from the (N–H⋯O = )_∞_ chains *via* rotation of the –CONHC_*n*_H_2*n*+1_ groups in Col_h_ phase. In addition, derivatives of *N*,*N*′-ditetradecyl-1,4-benzenedicarboxamide (2BC) and *N*,*N*′,*N*′′,*N*′′′,*N*′′′′-pentatetradecyl-1,2,3,4,5-benzenepentacarboxamide (5BC) were used to prepare ferroelectrics.^22^ Interestingly, a Col_h_ liquid crystalline material composed of *N*,*N*′,*N*′′,*N*′′′-tetratetradecyl-1,3,6,8-pyrenetetracarboxamide also formed a ferroelectric material with fluorescent and current switching properties.^23,24^ The 1D hydrogen-bonding molecular assembly and its dynamic behaviour are essential to the ferroelectric behaviour of the π-molecular system bearing multiple –CONHC_*n*_H_2*n*+1_ chains. Improved design of amide-type hydrogen-bonding has the potential to form low-dimensional hierarchical molecular assemblies such as rings, tubes, chains, and layer structures.

Additional hydrogen-bonding sites such as –OH, –NH_2_, and –COOH are usually introduced into molecular structures to form the low-dimensional molecular assemblies.^25–29^ Well-known hydrogen-bonding simple benzene carboxylic acid derivatives of trimesic acid can form two types of O–H⋯O = hydrogen-bonding molecular assemblies: a six-fold 2D hexagonal layer and an infinite 1D zig-zag chain, which can be controlled by crystallization conditions. On the other hand, terephthalic and isophthalic acids typically form 1D linear and 1D zig-zag type O–H⋯O = hydrogen-bonding molecular assemblies, respectively.^6–8^ It can be reasonably expected that these benzene carboxylic acid derivatives can act as effective platforms to construct various molecular assemblies in a flexible and rational way by introducing additional hydrogen-bonding group. For instance, an interesting ring-shaped molecular assembly was reported using an isophthalic acid (IP) derivative bearing a hydrophobic –OC_*n*_H_2*n*+1_ chain at the 5-position, which formed a hydrogen-bonding O–H⋯O = hexamer structure at *n* < 12, and each hexamer ring was isolated in the absence of interactions between the hexamers.^30–32^ In contrast, 1D zig-zag hydrogen-bonding structures have been observed in long alkyl chain compounds with *n* > 12, where the much longer alkyl chains enhanced hydrophobic interactions and stabilized the interdigitate lamellar-type molecular assembly structure. Although interesting ring-shaped hydrogen-bonding hexamer assemblies have been obtained by introducing alkoxy group into IP derivative, additional introduction of amide-type N–H⋯O = hydrogen-bonding interaction at –CONHC_*n*_H_2*n*+1_ chain has a potential to form various kinds of low-dimensional hierarchical molecular assembly structures.

**Scheme 1:**
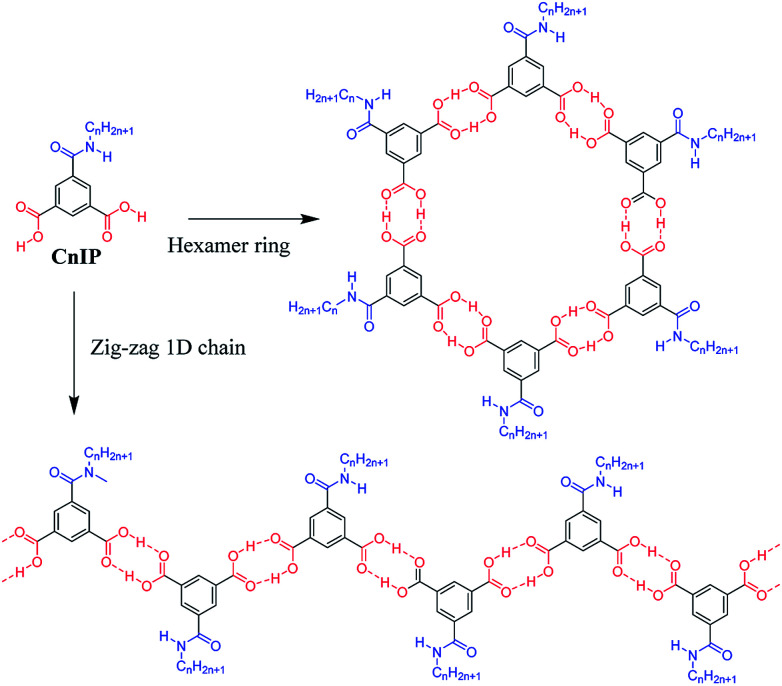
Two kinds of primary hydrogen-bonding interactions (red O–H⋯O interaction) to form the ring-hexamer (CnIP)_6_ and the zig-zag 1D chain (CnIP)_∞_. The diameter of the inner pore is approximately 1.1 nm for the hexamer (CnIP)_6_. Further secondary N–H⋯O = hydrogen-bonding interactions (blue N–H⋯O = ) assembled each hexamer and/or chain to form tubular and lamellar type molecular assemblies, respectively.

Herein, we designed a hydrogen-bonding IP derivative bearing an additional hydrogen-bonding –CONHC_*n*_H_2*n*+1_ chain to achieve low-dimensional molecular assemblies, which will fabricate different types of molecular assemblies from –OC_*n*_H_2*n*+1_ substituted IP derivative. Four kinds of amphiphilic IP derivatives bearing a different alkyl chain length of –CONHC_*n*_H_2*n*+1_, C6IP (*n* = 6), C10IP (*n* = 10), C14IP (*n* = 14), and C18IP (*n* = 18), were synthesized and corresponding molecular assembly behaviours were studied systematically. Different from the IP derivative with an –OC_*n*_H_2*n*+1_ chain, the hydrogen-bonding –CONHC_*n*_H_2*n*+1_ chain in CnIP can provide additional –N–H⋯O = hydrogen-bonding interactions to form high-order molecular assembly structures (Scheme 1). The O–H⋯O = hydrogen-bonding ring-hexamers are connected by six amide-type N–H⋯O = hydrogen-bonding interactions along the π-stacking direction of the hexamer, resulting in a tubular hierarchical molecular assembly with an inner pore diameter of ∼1.1 nm. Since the outer surface and inner pore of the tubular assembly are hydrophobic and hydrophilic, respectively, the inner hydrophilic pore can capture hydrophilic species such as H_2_O and various ions. Another possible molecular assembly is a zig-zag type O–H⋯O hydrogen-bonding 1D assembly, which are connected by amide-type N–H⋯O = hydrogen-bonding interactions along the direction normal to the 1D chain. These chains are formed in a similar manner as the lamellar-type 2D molecular assembly structure with alternating arrangement of the hydrogen-bonding layer and hydrophobic alkyl chains. Accordingly, both organogel and liquid crystal states were formed in the assembly system *via* additional intermolecular N–H⋯O = amide-type hydrogen-bonding interactions in a prime IP molecular core. Moreover, the liquid crystalline phase was modulated by the gelation process of C18IP. Pure unhydrated state formed the lamellar type liquid crystalline phase, whereas the xerogel state of (C18IP)_6_·(H_2_O)_*n*_ from C_2_H_5_OH–H_2_O formed the tubular structure and hexagonal columnar liquid crystalline phase. The gelation ability was directly associated with the formation of lamellar and/or hexagonal columnar phases. The thermal stability, organogelation ability, liquid crystal formation, phase transition behaviour, and ion inclusion properties of the prepared assemblies were systematically examined.

## Experimental section

### Preparation of CnIP

**Scheme 2:**
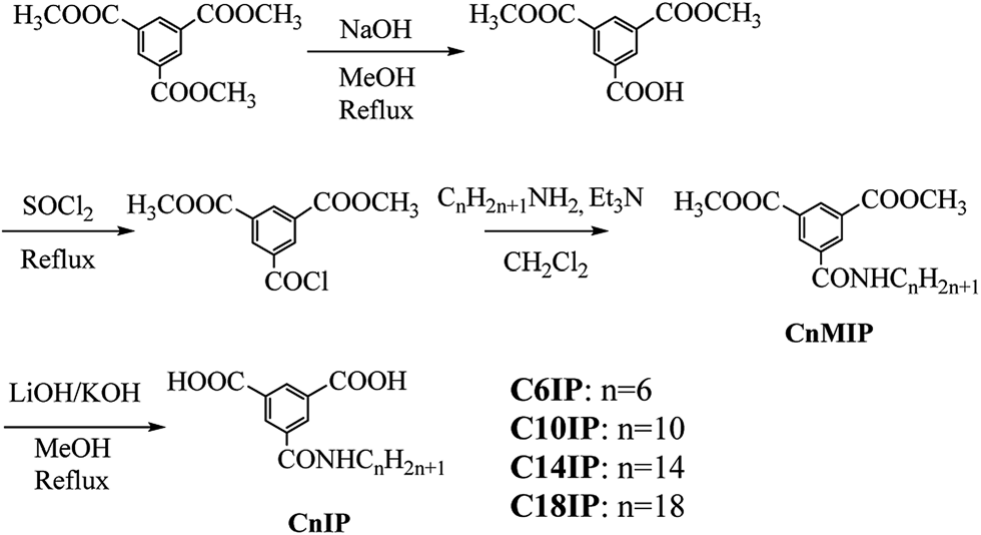
Synthetic procedure for the CnIP derivatives.

Commercially available reagents were used without further purification, and dry triethylamine was obtained by distillation from KOH. The preparation of CnIP (*n* = 6, 10, 14, and 18) was performed by a 4-step procedure starting from 1,3,5-benzenetricarboxylic acid trimethyl ester (Scheme 2). Benzene-1,3,5-tricarboxylic acid dimethyl ester was prepared following the literature.^33^ Benzene-1,3,5-tricarboxylic acid trimethyl ester (5.00 g, 7.93 mmol) and NaOH (790 mg, 19.8 mmol) were dissolved in CH_3_OH (175 mL) and refluxed for 12 h. The reaction mixture was diluted with water (500 mL), and washed with diethyl ether (3 × 250 mL). The aqueous phase was acidified to pH ∼ 1 with 5% hydrochloric acid and extracted with diethyl ether (3 × 250 mL). The extracted organic layers were dried over MgSO_4_ and concentrated under vacuum to afford benzene-1,3,5-tricarboxylic acid dimethyl ester(3.83 g) with a yield of 81%. ^1^H NMR (400 MHz, DMSO-*d*_6_): 8.60–8.65 (m, 3H, Ar–H), 3.93 (s, 6H, –OCH_3_).

### C6IP

Benzene-1,3,5-tricarboxylic acid dimethyl ester (2.10 g, 8.81 mmol) in SOCl_2_ (80 mL, 1.10 mol) was refluxed for 20 h. After cooling the reaction mixture to room temperature, the excess SOCl_2_ was removed under vacuum. The resultant acid chloride was dissolved in dry CH_2_Cl_2_ (40 mL), and hexylamine (1.32 mL, 10.1 mmol) was slowly added dropwise over 10 min followed by the addition of triethylamine (1.28 mL, 9.24 mmol). The mixture was subsequently stirred for 18 h at room temperature. The resultant solution was washed 3 times with aqueous 1 M HCl and brine, and then dried over Na_2_SO_4_. Evaporation of CH_2_Cl_2_ under reduced pressure yielded a white precipitate, which was recrystallized from hexane : AcOEt (2 : 5). A total of 1.52 g of *N*-hexyl-3,5-bis(methoxylcarbonyl)benzamide (C6MIP) was obtained with a yield of 56%. ^1^H NMR (400 MHz, CDCl_3_): *δ* 0.90 (t, *J* = 7.1 Hz, 3H, –CH_3_), 1.30–1.45 (m, 6H, alkyl), 1.62–1.69 (m, 2H, –NHCH_2_CH_2_), 3.45–3.52 (m, 2H, –NHCH_2_CH_2_), 3.98 (s, 6H, 2Ar-COOCH_3_), 6.26 (s, 1H, NH), 8.61 (d, *J* = 1.6 Hz, 2H, ArH), 8.80 (t, *J* = 1.6 Hz, 1H, ArH).

A solution of C6MIP (1.52 g, 4.73 mmol) in CH_3_OH (25 mL) was added to a solution of KOH (2.91 g, 51.8 mmol) in water (20 mL), and the resulting solution was refluxed for 2 h. After evaporation of CH_3_OH under vacuum, the reaction mixture was diluted with H_2_O (300 mL) and aqueous HCl (1 M) until the pH reached 1. The resulting suspension was extracted by AcOEt and dried over Na_2_SO_4_. Removal of solvent under vacuum afforded a crude product, which was recrystallized twice from CH_3_OH : H_2_O (4 : 1) to give C6IP (880 mg) with a yield of 63%. Mp = 279–280 °C. ^1^H NMR (400 MHz, DMSO-*d*_6_): 0.87 (t, *J* = 6.8 Hz, 3H, –CH_3_), 1.23–1.36 (m, 6H, alkyl), 1.48–1.58 (m, 2H, –NHCH_2_CH_2_), 3.23–3.31 (m, 2H, –NHCH_2_), 8.56 (t, *J* = 1.6 Hz, 1H, ArH), 8.63 (d, *J* = 1.6 Hz, 2H, ArH), 8.88 (t, *J* = 5.6 Hz, 1H, –NH), 13.50 (s, 2H, –(C=O)–OH). IR: *ν* (cm^−1^): 3287 (N–H stretch), 1688 (C=O of COOH), 1634 (C=O of CONH), 1544 (C–N). Elemental analysis: calculated for C_15_H_19_NO_5_: C, 61.42; H, 6.53; N, 4.78. Found: C, 61.25; H, 6.64; N, 4.82. High resolution mass spectrometry (HRMS) fast atom bombardment (FAB): calculated for C_15_H_20_NO_5_*m*/*z* 294.1341 [(M + H)^+^], found *m*/*z* 294.1339.

Preparations of C10IP, C14IP, and C18IP were performed by the similar procedure to that of C6IP using the corresponding alkylamines (see the ESI† for detail).

#### C10MIP

Yield 928 mg, 29%. ^1^H NMR (400 MHz, CDCl_3_): *δ* 0.88 (t, *J* = 6.8 Hz, 3H, –CH_3_), 1.20–1.45 (m, 14H, alkyl), 1.58–1.69 (m, 2H, –NHCH_2_CH_2_), 3.45–3.52 (m, 2H, –NHCH_2_CH_2_), 3.98 (s, 6H, 2Ar–COOCH_3_), 6.22 (br s, 1H, NH), 8.61 (d, *J* = 1.6 Hz, 2H, ArH), 8.79 (t, *J* = 1.6 Hz, 1H, ArH).

#### C10IP

Yield 760 mg, 63%. Mp = 245–246 °C. ^1^H NMR (400 MHz, DMSO-*d*_6_): *δ* 0.84 (t, *J* = 6.8 Hz, 3H, –CH_3_), 1.16–1.35 (m, 14H, alkyl), 1.48–1.59 (t, *J* = 6.8 Hz, 2H, –NHCH_2_CH_2_), 3.27 (q, *J* = 6.5 Hz, 2H, –NHCH_2_), 8.56 (t, *J* = 1.6 Hz, 1H, ArH), 8.63 (t, *J* = 1.6 Hz, 2H, ArH), 8.87 (t, *J* = 5.2 Hz, 1H, –NH), 13.50 (s, 2H, –(C=O)–OH). IR: *ν* (cm^−1^): 3300 (N–H stretch), 1720 and 1695 (C=O of COOH, C=O stretching vibration of the free, non-hydrogen bonded and laterally hydrogen-bonded COOH groups, respectively), 1640 (C=O of CONH), 1544 (C–N). Elemental analysis: calculated for C_19_H_27_NO_5_: C, 65.31; H, 7.79; N, 4.01. Found: C, 65.28; H, 7.91; N, 4.00. HRMS (FAB): calculated for C_19_H_28_NO_5_*m*/*z* 350.1967 [(M + H)^+^], found *m*/*z* 350.1972.

#### C14MIP

Yield 1.08 g, 73%. ^1^H NMR (400 MHz, CDCl_3_): *δ* 0.88 (t, *J* = 6.4 Hz, 3H, CH_3_), 1.21–1.44 (m, 22H, alkyl), 1.59–1.69 (m, 2H, –NHCH_2_CH_2_), 3.44–3.53 (m, 2H, –NHCH_2_CH_2_), 3.98 (s, 6H, 2Ar–COOCH_3_), 6.21 (br s, 1H, NH), 8.61 (d, *J* = 1.6 Hz, 2H, ArH), 8.79 (t, *J* = 1.6 Hz, 1H, ArH).

#### C14IP

Yield 646 mg, 64%. Mp: decomposed at above 241 °C. ^1^H NMR (400 MHz, DMSO-*d*_6_): *δ* 0.85 (t, *J* = 6.8 Hz, 3H, CH_3_), 1.18–1.35 (m, 22H, alkyl), 1.48–1.58 (m, 2H, CONHCH_2_CH_2_), 3.29 (m, 2H, CONHCH_2_), 8.56 (t, *J* = 1.6 Hz, 1H, ArH), 8.62 (s, 2H, ArH), 8.85 (br t, *J* = 4.8 Hz, 1H, NH), 13.47 (br s, 2H, (C=O)–OH). IR: *ν* (cm^−1^): 3300 (N–H stretch), 1720 and 1695 (C=O of COOH, C=O stretching vibration of the free, non-hydrogen bonded and laterally hydrogen-bonded CO_2_H groups, respectively), 1637 (C=O of CONH), 1537 (C–N). Elemental analysis calculated for C_23_H_35_NO_5_: C, 68.12; H, 8.70; N, 3.45, found: C, 68.37; H, 8.96; N, 3.57. HRMS (FAB): calculated for C_23_H_36_NO_5_*m*/*z* 406.2593 [(M + H)^+^], found *m*/*z* 406.2594.

#### C18MIP

Yield 2.40 g, 58%. ^1^H NMR (400 MHz, CDCl_3_): *δ* 0.85 (t, *J* = 6.8 Hz, 3H, CH_3_), 1.21–1.44 (m, 30H, alkyl), 1.60–1.70 (m, 2H, –NHCH_2_CH_2_), 3.45–3.52 (m, 2H, –NHCH_2_CH_2_), 3.98 (s, 6H, 2Ar–COOCH_3_), 6.22 (s, 1H, NH), 8.61 (d, *J* = 1.6 Hz, 2H, ArH), 8.80 (t, *J* = 1.6 Hz, 1H, ArH).

#### C18IP

Yield 1.77 g, 83%. Mp: decomposed at above 231 °C. ^1^H NMR (400 MHz, DMSO-*d*_6_): *δ* 0.85 (t, *J* = 6.8 Hz, 3H, –CH_3_), 1.17–1.35 (m, 30H, alkyl), 1.47–1.58 (m, 2H, –NHCH_2_CH_2_), 3.23–3.29 (m, 2H, –NHCH_2_), 8.56 (t, *J* = 1.6 Hz, 1H, ArH), 8.63 (s, 2H, ArH), 8.87 (t, *J* = 5.5 Hz, 1H, –NH), 13.47 (br s, 2H, –(C=O)–OH). IR: *ν* (cm^−1^): 3303 (N–H stretch), 1720 and 1695 (C=O of COOH, C=O stretching vibration of the free, non-hydrogen bonded and laterally hydrogen-bonded CO_2_H groups, respectively), 1637 (C=O of CONH), 1537 (C–N). Elemental analysis: calculated for C_27_H_43_NO_5_: C, 70.25; H, 9.39; N, 3.03. Found: C, 69.97; H, 9.45; N, 3.15. HRMS (FAB): calculated for C_27_H_44_NO_5_*m*/*z* 462.3219 [(M + H)^+^], found *m*/*z* 462.3219.

### Preparation of organogels

Temperature and solvent dependent gelation behaviors were examined following in the literature.^34^ A series of commonly used solvent such as CH_3_OH, C_2_H_5_OH, acetone, THF, CH_3_CN, AcOEt, toluene, and H_2_O was screened for the organogel formation of CnIP. The gelation behavior was not confirmed in each pure solvent. After the screening in the mixed solvent system, the organogels of CnIP were observed in C_2_H_5_OH–H_2_O. CnIP in hot C_2_H_5_OH–H_2_O with concentration of 10 mM was gradually cooled down to room temperature, and the minimum volume percentage of H_2_O to induce the organogels at 10 mM was summarized in Table 1.

**Table 1:** Formation of OG and LC states of CnIP derivatives at *n* = 6, 10, 14, and 18

	C6IP	C10IP	C14IP	C18IP
Formation of OG^a^	—f	○^e^	○^e^	○^e^
H_2_O (v/v%) in 10 mM solution^b^	—	50	40	30
Xerogels^c^	—	—	○^e^	○^e^
H_2_O (wt%) of xerogel^c^	—	—	3.7–7.2%	3.3–7.3%
H_2_O (wt%)% of crystal	0.0	0.0	0.0	0.0
Type of LC phase^d^	—	—	Col_h_	Col_h_ and L_a_

a OG formation was evaluated in a 10 mM solution of CnIP in C_2_H_5_OH–H_2_O (v/v = volume percentage of H_2_O) at 300 K.

b Volume percentage of H_2_O for OG formation of CnIP at *ca.* fixed concentration of 10 mM in C_2_H_5_OH.

c Xerogels were obtained by vacuum evaporation of the OG and the weight percentage of H_2_O was determined by TG analyses.

d LC phases of Col_h_ and L_a_ were discotic hexagonal columnar and lamella phases, respectively. Solvent loss of the C10IP xerogel was not obtained in the vacuum drying process at room temperature, and OG state was transformed to a crystalline solid.

e The notation of “○”represented the formation of OG or xerogel states at a fixed concentration of 10 mM in C_2_H_5_OH–H_2_O.

f OG states were not observed in C_2_H_5_OH–H_2_O at v/v range from 100/0 to 0/100.

### Physical measurements

Infrared spectroscopy (IR; Thermo Fisher Scientific Nicolet 6700, 400–4000 cm^−1^) measurements were conducted with a resolution of 4 cm^−1^ using KBr pellets. Thermogravimetry-differential thermal analyses (TG-DTA) were conducted using a thermal analysis station (Rigaku Thermo plus TG8120) with Al_2_O_3_ as a reference from 293 to 600 K with a heating rate of 5 K min^−1^ under a nitrogen atmosphere. Temperature-dependent powder pattern X-ray diffraction (XRD) data were collected using a diffractometer (Rigaku Rint-Ultima III) with Cu Kα (*λ* = 1.54187 Å) radiation. Scanning electron microscopy (SEM; JEOL JSEM-5400F) and atomic force microscopy (AFM; JEOL JSPM-5200) were performed on highly ordered pyrolytic graphite (HOPG) and mica substrates, respectively. Acceleration voltages of 5 or 10 kV under a vacuum of less than 10^−4^ Pa was used for the SEM measurements. Commercially available Si cantilevers with a force constant of 4.5 N m^−1^ were used for the AFM measurements.

## Results and discussion

### Formation of organogels

**Fig. 1:**
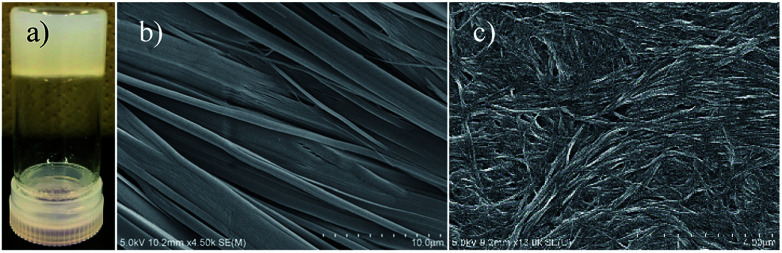
Optical microscopy and SEM images of the OG, CS, and XG states. (a) Cloudy OG state of C14IP in C_2_H_5_OH–H_2_O. SEM image of (b) the CS state of unhydrated C18IP and (c) the XG state of C18IP on silicon.

The CnIP molecules (*n* = 6, 10, 14, and 18) possess two kinds of hydrogen-bonding sites; two hydrophilic –COOH groups and one hydrophobic –CONHC_*n*_H_2*n*+1_ chain, resulting in amphiphilic properties. The hydrophobic character of the CnIP derivatives was enhanced with increasing alkyl chain length (*n*) from C6IP, C10IP, C14IP, to C18IP, which affected the molecular assembly structures of the organogel (OG) and liquid crystal (LC). The CnIP molecules were soluble in CH_3_OH, C_2_H_5_OH, acetone, and THF, whereas only slightly soluble in H_2_O, CH_3_CN, AcOEt, and toluene. Among the four CnIP derivatives, C10IP, C14IP, and C18IP can form OGs in the mixed solvent system of C_2_H_5_OH (CH_3_OH)–H_2_O (Fig. 1a) at a fixed concentration of 10 mM. Table 1 summarizes the formation of OGs in C_2_H_5_OH–H_2_O and LCs of the CnIP derivatives. C6IP with the shortest alkyl chain did not form the OG or LC state due to insufficient hydrophobicity. The formation of an OG is closely related to the formation of 1D fibrous molecular assemblies and their 3D entanglements.

Therefore, the formation of 1D hydrogen-bonding tubular assembly was consistent with the formation of an OG. Among the poor solvents, H_2_O was essential for the formation of the OG of C10IP, C14IP, and C18IP, and C_2_H_5_OH and/or CH_3_OH were also needed. Although we tried to prepare OG state from a variety of solvent such as THF, CH_3_CN, AcOEt, and toluene, the addition of H_2_O in C_2_H_5_OH achieved the corresponding organogel states. The thermal stability of the OG decreased in the order of C18IP, C14IP, to C10IP, and crystalline powder coexisted with the OG in the shorter chain C10IP molecule. Therefore, crystallinity was increased by decreasing the alkyl chain length. The solubility of CnIP in C_2_H_5_OH decreased in the order of C6IP, C10IP, C14IP, to C18IP, and the minimum H_2_O content required for the formation of the respective OGs also decreased in the same order (Table 1 and Fig. S1†). The volume percentages of H_2_O to form stable OGs at 20, 10, 5, and 2.5 mM solutions of C14IP in C_2_H_5_OH were approximately 20, 30, 40, and 50%, respectively.

### Hydrated xerogels and unhydrated crystals

The formation of an OG was not observed in the shortest alkyl chain C6IP derivative, whereas the OG state of C10IP was unstable at 300 K due to the coexistence of OG and crystalline solid (CS) states. Both C14IP and C18IP derivatives can form stable and uniform OG states in C_2_H_5_OH–H_2_O mixed solvents in the absence of CS at approximately 300 K. Two kinds of molecular assembly states of the xerogel (XG) and CS were obtained for C14IP and C18IP (Fig. S2†) by changing the solvent system. The XG state was obtained by vacuum drying from the OG state in C_2_H_5_OH–H_2_O, whereas the CS was obtained by recrystallization from CH_3_OH, C_2_H_5_OH, or acetone in the absence of H_2_O. The presence of H_2_O was a necessary condition to form both the OG and XG states. Fig. 1a shows a cloudy OG state of C14IP in C_2_H_5_OH–H_2_O at a fixed concentration of 10 mM. The microscale morphologies of the CS and XG states of C18IP on silicon differed significantly in the SEM images. Fig. 1b and c show the SEM images of the CS and XG states of C18IP. The morphology of the CS state has a clear edge with a flat crystal surface (Fig. 1b), whereas the 3D entanglement network of each microfiber was observed in the XG state of C18IP (Fig. 1c). The latter 3D entangled microstructure was a prerequisite for the formation of the OG state. Differences in the microscale morphologies of the XG and CS are associated with microscale hydrogen-bonding interactions.

**Fig. 2:**
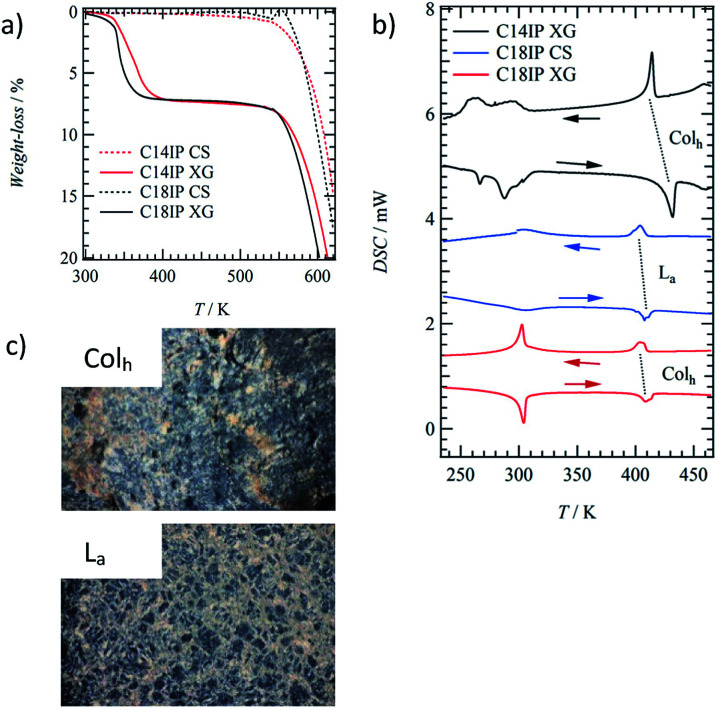
Thermal properties of the XG states of (C14IP)_6_·(H_2_O)_*n*_ and (C18IP)_6_·(H_2_O)_*n*_ and CS state of C18IP. (a) TG diagrams of the XG (solid line) and CS (dashed line) states of C14IP (red) and C18IP (black). (b) The DSC diagrams for XG of (C14IP)_6_·(H_2_O)_*n*_, (C18IP)_6_·(H_2_O)_*n*_, and CS of C18IP. (c) POM images of the Col_h_ and L_a_ phases of (C18IP)_6_·(H_2_O)_*n*_ and C18IP at 450 K.

The results of the TGA of the XG and CS states of C14IP and C18IP differed to significant degree (Fig. 2a). Although the CS state exhibited high thermal stability up to 520 K, as indicated by the absence of weight-loss, the XG state showed weight-loss at approximately 400 K corresponding to the desorption of H_2_O molecules. The magnitude of the weight-losses for the XG states of (C14IP)_6_·(H_2_O)_*n*_ and (C18IP)_6_·(H_2_O)_*n*_ at 400 K were 3.7–7.2 and 3.3–7.3%, respectively, corresponding to 5 ≤ *n* ≤ 12 (Fig. S3†). It should be noted that the amount of H_2_O molecules may be influenced by environmental humidity during the vacuum drying process, accounting for the deviation in H_2_O content. The presence of H_2_O is a requirement for the formation of the OG state, where the hydrophilic H_2_O molecules stabilize the 1D fibrous molecular assemblies. Since the microfibrous morphology of the XG state was maintained even under vacuum during the SEM measurements, it can be concluded that the 1D fibrous molecular assemblies of C14IP and C18IP were stabilized by intermolecular hydrogen-bonding interactions.

### Liquid crystalline property

The LC phases were not observed in the short alkyl chain compounds of C6IP and C10IP with direct decomposition at approximately 550 K (Fig. S4†). In contrast, both fluidic and birefringent behaviours of (C14IP)_6_·(H_2_O)_*n*_, (C18IP)_6_·(H_2_O)_*n*_, and unhydrated C18IP were observed in the polarized optical microscopy (POM) images under the cross-Nicol optical arrangement by increasing the temperature to 420 K.

The XG and CS states of C14IP and C18IP showed different phase transition behaviours in the DSC diagrams. The endothermic peaks of the XG state of (C14IP)_6_·(H_2_O)_*n*_ were observed at approximately 270 and 300 K during the heating process, which corresponded to H_2_O melting within the molecular assembly and were also observed in the exothermic peaks during cooling (Fig. 2b). The reversible endothermic and exothermic peaks at approximately 420 K corresponded to the phase transition from solid (S) to LC phase (black DSC chart in Fig. 2b). However, the phase transition from the LC to the isotopic liquid (IL) phase was not observed in the DSC of (C14IP)_6_·(H_2_O)_*n*_ due to decomposition at approximately 540 K. Similar phase transition behaviour was observed in the XG state of (C18IP)_6_·(H_2_O)_*n*_, where a reversible S-LC phase transition peak was observed at approximately 410 K (red DSC chart in Fig. 2b). Although the phase transition behaviour of unhydrated C14IP was fundamentally similar to that of the hydrated (C14IP)_6_·(H_2_O)_*n*_, the CS state of unhydrated C18IP exhibited a reversible S–LC phase transition peak at approximately 410 K (blue DSC chart in Fig. 2b). The XG and CS states of (C18IP)_6_·(H_2_O)_*n*_ and C18IP formed different LC phases formed from different molecular assembly structures. Fig. 2c shows the POM images of the two mesophases derived from the XG and CS states of (C18IP)_6_·(H_2_O)_*n*_ and C18IP at 450 K. Although both POM textures resembled each other, a striped pattern was observed in the texture of the CS state. The molecular assembly structure within the LC phase is affected by the alkyl chain length (*n*) of the –CONHC_*n*_H_2*n*+1_ group.

**Fig. 3:**
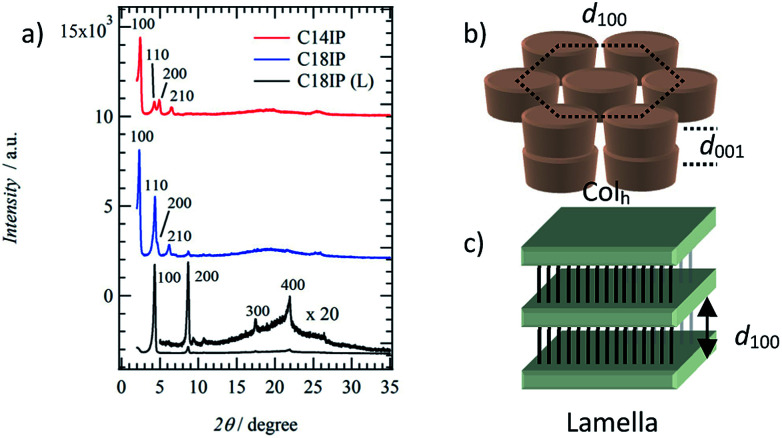
Molecular assembly structures of the LC phases. (a) The XRD patterns of the Col_h_ phase of (C14IP)_6_·(H_2_O)_*n*_ at 450 K (red), (C18IP)_6_·(H_2_O)_*n*_ at 480 K (blue), and L_a_ phase of C18IP at 460 K (black). Schematic packing structures and lattice periodicities of *d*_100_ and *d*_001_ for (b) Col_h_ and (c) L_a_ phases.

To identify the molecular assembly structures of the LC phases, the XRD patterns of the two types of LC phases of (C14IP)_6_·(H_2_O)_*n*_ (or (C18IP)_6_·(H_2_O)_*n*_) and unhydrated C18IP were compared. Fig. 3 summarizes the XRD patterns of the LC phases of (C14IP)_6_·(H_2_O)_*n*_, (C18IP)_6_·(H_2_O)_*n*_, and C18IP. Typical XRD patterns of (C14IP)_6_·(H_2_O)_*n*_ at 450 K were consistent the diffraction pattern of the discotic hexagonal columnar (Col_h_) LC phase with a *d*_100_ = 3.62 nm,^34–36^ where the diffraction peaks at 2*θ* = 2.44, 4.27, 4.91, and 6.56° were consistent with the index values of *d*_100_, *d*_110_, *d*_200_, and *d*_210_, respectively. In the large angle region, two broad diffraction peaks around 2*θ* = 20 and 25° could be assigned to the melting state of the alkyl chains and average interplanar distance (*d*_001_) along the π-stacking direction within a column. Almost identical XRD patterns were observed in LC phase of (C18IP)_6_·(H_2_O)_*n*_ at 480 K, which was also consistent with the Col_h_ phase of (C14IP)_6_·(H_2_O)_*n*_. A periodicity of *d*_100_ = 3.84 nm for (C18IP)_6_·(H_2_O)_*n*_ was approximately 0.22 nm longer than that of (C14IP)_6_·(H_2_O)_*n*_ (*d*_100_ = 3.62 nm). The *d*_100_-spacing for the Col_h_ phases of (C14IP)_6_·(H_2_O)_*n*_ and (C18IP)_6_·(H_2_O)_*n*_ was approximately 40 and 45% smaller than the maximum molecular lengths of 5.98 and 6.98 nm for the (C14IP)_6_ and (C18IP)_6_ hexamers, respectively, assuming an all-*trans* –CONHC_*n*_H_2*n*+1_ conformation, consistent with the melting state of the alkyl chains in the Col_h_ phase. It is worth to mention that the three-dimensional molecular assemblies of CnIP is observed even in fluid high temperature LC phase. Similar molecular assemblies of alkoxyisophthalic acid derivative in the absence of additional hydrogen-bonding –CONHC_*n*_H_2*n*+1_ unit did not form the mesophase by a direct solid-isotropic liquid phase transition,^31^ which were completely different from the phase transition behaviors of C14IP and C18IP. This difference is clearly accounted for the role of amide-type hydrogen-bonding interaction, which significantly connects each two-dimension hexameric ring structures to form tubular assembly through the strong amide-type hydrogen-bonding interaction of C14IP and C18IP. As a result, the corresponding tubular molecular assemblies were stabilized to exhibit LC phase and organogel state. Interestingly, the XRD pattern of the LC phase of unhydrated C18IP was not consistent with the formation of the Col_h_ phase. Highly ordered diffraction peaks at 2*θ* = 4.31, 8.68, 17.45, and 21.91° at *T* = 460 K were consistent with the index values of *d*_100_, *d*_200_, *d*_300_, and *d*_400_, suggesting a layered molecular assembly structure with an interlayer spacing of *d*_001_ = 2.05 nm. Therefore, the lamella (L_a_) type LC phase is formed during the heating of unhydrated C18IP.^35–37^ Infinite O–H⋯O = hydrogen-bonding chains were assembled to form a hydrogen-bonding 2D sheet through the interchain N–H⋯O = hydrogen-bonding interactions. The interlayer spacing of *d*_100_ = 2.05 nm in the L_a_ phase was 0.74 nm shorter than the maximum length of C18IP assuming an all-*trans* conformation of –CONHC_18_H_37_.

The H_2_O molecules play a key role in determining the type of LC phase (Col_h_ and/or L_a_). It should be noted that the hydrated (C14IP)_6_·(H_2_O)_*n*_ and (C18IP)_6_·(H_2_O)_*n*_ primarily formed the Col_h_ phase and unhydrated C18IP adopted the L_a_ phase. In the vibrational spectra (Fig. S6 and S7†), both the –OH and –NH stretching energies of *ν*^a^_O–H_ of the –COOH groups and of *ν*^a^_N–H_ of –CONH– for different molecular assemblies of the XG and CS states were observed at 3080 and 3302 cm^−1^, respectively. This suggests the formation of intermolecular O–H⋯O = and N–H⋯O = hydrogen-bonding interactions. In hydrated (C18IP)_6_·(H_2_O)_*n*_, amide-I and -II bands were observed at 1637 and 1599 cm^−1^, respectively, indicating the formation of intermolecular N–H⋯O = hydrogen-bonding interactions.^38^ On the other hand, the amide-I and -II bands of unhydrated C18IP were observed at 1616 and 1582 cm^−1^, respectively. Since the formation of strong hydrogen-bonding interactions usually results in a red-shift of the vibrational band, it can be concluded that the strength of hydrogen-bonding interaction of unhydrated C18IP was stronger than that of the hydrated (C18IP)_6_·(H_2_O)_*n*_. This corresponds to a much denser packing structure of the former state due to its high crystallinity. There was no significant difference in the vibrational spectra of the unhydrated C14IP and hydrated (C14IP)_6_·(H_2_O)_*n*_ (Fig. S7†), which formed a similar packing structure in both LC phases.

**Scheme 3:**
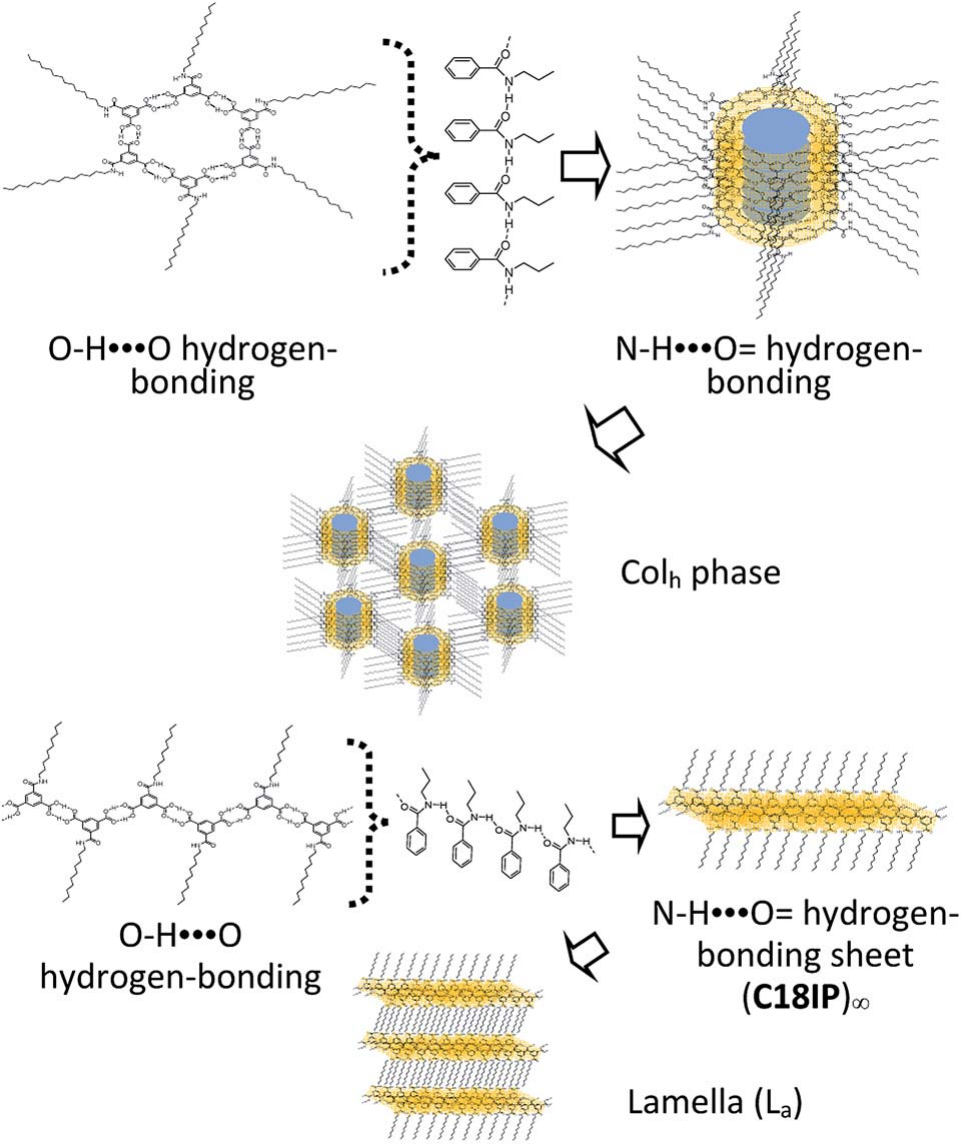
Formation of the hierarchical molecular assembly structures of C18IP to form the Col_h_ (upper) and L_a_ phases (lower).

The most widely accepted molecular assembly structure of (C18IP)_6_·(H_2_O)_*n*_ in the Col_h_ phase is a tubular ring-type structure, where the O–H⋯O = hydrogen-bonding hexamer-rings of (C18IP)_6_ are assembled to form a π-stacking tubular structure through intermolecular N–H⋯O = hydrogen-bonding interactions along the tube. The hydrophilic pore with a diameter of ∼1.1 nm was observed on the inner side of the ring-shaped (C18IP)_6_ hexamer, which was filled by 5–12 hydrophilic H_2_O molecules in the XG state. Assuming the π-stacking distance of (C18IP)_6_ is *d*_001_ = 3.8 Å in the Col_h_ phase and H_2_O molecular volume of ∼30 Å^3^, the hydrophilic volume available inside the pore is 361 Å^3^ per (C18IP)_6_ unit, consistent with the occupied volume of 360 Å^3^ for the 12 H_2_O molecules. The presence of H_2_O inside the tubular pore was also consistent with the TG and DSC analyses, formation of the Col_h_ phase, and XRD patterns. On the other hand, the outer surface of the tubular molecular assembly was covered by hydrophobic –CONHC_*n*_H_2*n*+1_ chains (Scheme 3).

Two types of LC phases (Col_h_ and L_a_) were only observed in unhydrated C18IP. In the L_a_ phase, each C18IP molecule formed one zig-zag O–H⋯O = hydrogen-bonding chain as a primary assembly structure. Secondary intermolecular N–H⋯O = hydrogen-bonding interactions generated the 2D molecular assembly, where the hydrophobic –CONHC_18_H_37_ chains were elongated along the direction normal to the hydrogen-bonding sheet to form a lamella-type molecular assembly in the absence of H_2_O.

### Dielectric responses and ion inclusion

**Fig. 4:**
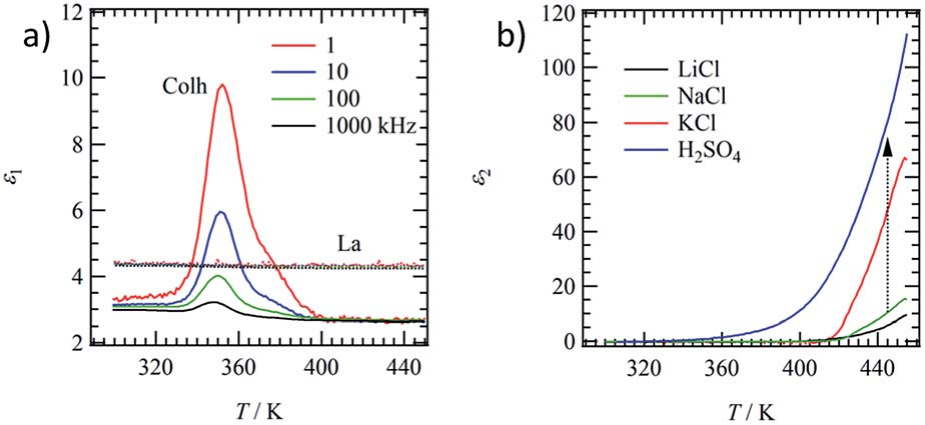
Dielectric responses of CnIP. (a) Temperature and frequency dependent *ε*_1_ of hydrated (Col_h_) and unhydrated (L_a_) states of C18IP. (b) Temperature dependent *ε*_2_ at *f* = 1 kHz of the tubular molecular assemblies of C14IP including LiCl (black line), NaCl (green line), KCl (red line), and H_2_SO_4_ (blue line).

Molecular motion in the Col_h_ and L_a_ phases were evaluated by the temperature- and frequency-dependent dielectric constants. The motional freedom of polar H_2_O molecules can be easily detected by dielectric spectroscopy.^39^Fig. 4a shows the temperature- and frequency-dependent real part dielectric constant, *ε*_1_, of hydrated (C18IP)_6_·(H_2_O)_*n*_ and unhydrated (C18IP)_∞_. The *ε*_1_-*T* plots of (C18IP)_6_·(H_2_O)_*n*_ showed a frequency dependent peak at 352 K, where the low frequency *ε*_1_ was significantly enhanced due to the slow molecular motion. Elimination of H_2_O in the TGA diagram was consistent with the dielectric anomaly at approximately 350 K. In contrast, the *ε*_1_ values of unhydrated (C18IP)_∞_ were temperature and frequency independent with a constant *ε*_1_ value of ∼4, completely different behavior to that of the hydrated (C18IP)_6_·(H_2_O)_*n*_. Therefore, the dielectric spectroscopies of hydrated (C18IP)_6_·(H_2_O)_*n*_ and unhydrated (C18IP)_∞_ confirmed the presence of H_2_O in the tubular assembly and its importance in the formation of different molecular assembly structures (Col_h_ and L_a_ phases).

Hydrophilic ionic pairs such as MX = LiCl, NaCl, KCl, and H_2_SO_4_ were introduced into the inner channel of tubular molecular assembly of (C14IP)_6_. Such channel formation based on tubular molecular assembly enables to achieve the transport property for various kinds of ions along the π-stacking and amide-type hydrogen-bonding direction.^40–43^ A mixing ratio of MX : (C14IP)_6_ = 1 : 1 can form the OG state in H_2_O–C_2_H_5_OH, which was subsequently dried under vacuum to form the XG state of (C14IP)_6_·(MX). From the elemental analyses (Table S1†), the formulae observed were (C14IP)_6_·(LiCl–H_2_O), (C14IP)_6_·(NaCl–H_2_O), (C14IP)_6_·(KCl–H_2_O), and (C14IP)_6_·(H_2_SO_4_–H_2_O). The formation of hexagonal columnar structure was confirmed by XRD showing typical hydrogen-bonding tubular molecular assembly structures. Therefore, the inner hydrophilic channel of the tubular molecular assembly of (C14IP)_6_ was filled with the corresponding hydrophilic MX species. The dielectric responses of (C14IP)_6_·(MX) differ according to MX species. Fig. 4b summarizes the imaginary part of the dielectric constant, *ε*_2_, of (C14IP)_6_·(MX) at a constant frequency of *f* = 1 kHz in the second heating process. Since the H_2_O molecules in the second heating process were completely removed from the tubular molecular assemblies, the intrinsic dielectric responses of the unhydrated (C14IP)_6_·(MX) were observed in the temperature dependent *ε*_2_. The *ε*_2_ values corresponded to the dielectric-loss and/or conductive component in a parallel RC-circuit. The *ε*_2_ values below 350 K were almost zero due to a lack of contribution from the conducting component. However, large *ε*_2_ differences were observed above 400 K according to the MX species present in the tubular channel. The *ε*_2_ values of (C14IP)_6_·(LiCl), (C14IP)_6_·(NaCl), (C14IP)_6_·(KCl), and (C14IP)_6_·(H_2_SO_4_) at 450 K were 7.5, 13, 60, and 100, respectively. The relatively high *ε*_2_ value of (C14IP)_6_·(H_2_SO_4_) originated from the protonic conductivity, and the magnitude of the *ε*_2_ values decreased in the order of H_2_SO_4_, KCl, NaCl, to LiCl. Large MX salts have a tendency to increase the *ε*_2_ values and the compatibility of pore diameter (∼1.1 nm) and size of the MX pair is essential in determining the *ε*_2_ values.

## Conclusions

Hydrophobic alkylamide chains (–CONHC_*n*_H_2*n*+1_) were introduced as an effective intermolecular N–H⋯O = hydrogen-bonding unit into a O–H⋯O hydrogen-bonding isophthalic acid derivative (CnIP). The molecular aggregation behaviours of the prepared derivatives were examined by changing the alkyl chain length from *n* = 6, 10, 14, to 18. The O–H⋯O hydrogen-bonding interactions at the two –COOH sites of the isophthalic acid derivatives could form two kinds of hydrogen-bonding structures; a 1D zig-zag chain and ring-shaped (CnIP)_6_ hexamer according to the parameter *n*. Ring-shaped O–H⋯O hydrogen-bonding hexamers were obtained in the OG state of (C14IP)_6_·(H_2_O)_*n*_ and (C18IP)_6_·(H_2_O)_*n*_, which were further assembled to a tubular molecular assembly *via* inter-hexamer N–H⋯O = hydrogen-bonding. The formation of the OG in H_2_O–C_2_H_5_OH was consistent with the fibrous 1D molecular assembly with a tubular structure. The hydrophilic inner pores of the 1D tubular assemblies of the XG states of (C14IP)_6_·(H_2_O)_*n*_ and (C18IP)_6_·(H_2_O)_*n*_ were occupied by H_2_O molecules, forming a thermotropic Col_h_ phase upon heating. In contrast, enhanced hydrophobic interactions in C18IP formed a lamella-type molecular assembly structure in the absence of H_2_O, where the 1D zig-zag O–H⋯O hydrogen-bonding chains interacted through additional N–H⋯O = hydrogen-bonding to form a 2D sheet assembly. No void space in the lamella phase was observed in the absence of H_2_O. On the other hand, the 1D hydrophilic pore with a diameter of 1.1 nm in the Col_h_ phase was suitable for H_2_O occupation and formation of an ion pair (MX = LiCl, NaCl, KCl, and H_2_SO_4_) inclusion environment. Slight modification of the hydrophobic interactions in the –CONHC_*n*_H_2*n*+1_ chains was essential for the formation of the tubular or lamella type hydrogen-bonding molecular assembly structures in simple alkylamide-substituted isophthalic acid derivatives. The hydrophilic inner channel of (CnIP)_6_ tubular assemblies can be used for molecular and ionic transport.

## Conflicts of interest

There are no conflicts to declare.

## Supplementary Material

RA-008-C8RA04077J-s001

## References

[cit1] The Amide Linkage, ed. A. Greenberg, C. M. Breneman and J. F. Liebman, Wiley-Interscience, NJ, 2003

[cit2] Peptide Solvation and H-Bonds, Advances in Protein Chemistry, ed. R. L. Baldwin and D. Baker, Elsevier Academic Press, Amsterdam, 2006, vol. 72

[cit3] Desiraju G. R. , Steiner T., The Weak Hydrogen Bond, Oxford University Press, NY, 1999

[cit4] Jeffrey G. A. , An Introduction to Hydrogen Bonding, ed. D. G. Truhlar, Oxford University Press, NY, 1997

[cit5] Steiner T. (2002). Angew. Chem., Int. Ed..

[cit6] Bailey M., Brown C. J. (1967). Acta Crystallogr..

[cit7] Alcala R., Martinez-Carreras S. (1972). Acta Crystallogr., Sect. B: Struct. Crystallogr. Cryst. Chem..

[cit8] Derissen J. L. (1974). Acta Crystallogr., Sect. B: Struct. Crystallogr. Cryst. Chem..

[cit9] Semmingsen D. (1973). Acta Chem. Scand..

[cit10] Duchamp D. J., Marsh R. E. (1969). Acta Crystallogr., Sect. B: Struct. Crystallogr. Cryst. Chem..

[cit11] Ermer O., Lindenberg L. (1991). Helv. Chim. Acta.

[cit12] Ermer O., Eling A. (1988). Angew. Chem., Int. Ed..

[cit13] Katrusiak A., Szafrańsk M. (1999). Phys. Rev. Lett..

[cit14] Szafrański M., Katrusiak A. (2004). J. Phys. Chem. B.

[cit15] Akutagawa T., Takeda S., Hasegawa T., Nakamura T. (2004). J. Am. Chem. Soc..

[cit16] Horiuchi S., Tokunaga Y., Giovannetti G., Picozzi S., Itoh H., Shimano R., Kumai R., Tokura Y. (2010). Nature.

[cit17] Horiuchi S., Kagawa F., Hatahara K., Kobayashi K., Kumai R., Murakami Y., Tokura Y. (2012). Nat. Commun..

[cit18] Sugita A., Suzuki K., Tasaki S. (2004). Chem. Phys. Lett..

[cit19] Sugita A., Suzuki K., Kubono A., Tasaki S. (2008). Jpn. J. Appl. Phys..

[cit20] Fitié C. F. C., Roelofs W. S. C., Kemerink M., Sijbesma R. P. (2010). J. Am. Chem. Soc..

[cit21] Fitié F. C. F. C., Roelofs W. S. C., Magusin P. C. M. M., Wübbenhorst M., Kemerink M., Sijbesma R. P. (2012). J. Phys. Chem. B.

[cit22] Shishido Y., Anetai H., Takeda T., Hoshino N., Noro S., Nakamura T., Akutagawa T. (2014). J. Phys. Chem. C.

[cit23] Anetai H., Wada Y., Takeda T., Hoshino N., Yamamoto S., Mitsuishi M., Takenobu T., Akutagawa T. (2015). J. Phys. Chem. Lett..

[cit24] Anetai H., Takeda T., Hoshino N., Araki Y., Wada T., Yamamoto S., Mitsuishi M., Tsuchida H., Ogoshi T., Akutagawa T. (2018). J. Phys. Chem. C.

[cit25] Gilli G. , Gilli P., The Nature of the Hydrogen Bond, Outline of a Comprehensive Hydrogen Bond Theory, Oxford Univ. Press, Oxford, 2009

[cit26] Supramolecular Assembly *via* Hydrogen Bonds I and II, ed. D. M. P. Mingos, Springer, Berlin, 2004

[cit27] González-Rodríguez D., Schenning A. P. H. J. (2011). Chem. Mater..

[cit28] Yang Y., Wang C. (2009). Chem. Soc. Rev..

[cit29] Zhang Y., Li Y., Liu W. (2014). Adv. Funct. Mater..

[cit30] Yang J., Marendaz J. L., Geib S. J., Hamilton A. D. (1994). Tetrahedron Lett..

[cit31] Enkelmann V., Valiyaveettil S., Miiessner G., Miillen K. (1995). Supramol. Sci..

[cit32] Pfaadt M., Moessner G., Pressner D., Valiyaveettil S., Boeffel C., Müllen K., Spiess H. W. (1995). J. Mater. Chem..

[cit33] Roosma J., Mes T., Leclère P., Palmans A. R. A., Meijer E. W. (2008). J. Am. Chem. Soc..

[cit34] Buerkle J. E., Rowan S. J. (2012). Chem. Soc. Rev..

[cit35] Kumar S. , Chemistry of Discotic Liquid Crystals, V. Percec, ed.; CRC Press, NY, 2011

[cit36] Collings P. J. , Hird M., Introduction to Liquid Crystals, Chemistry and Physics, eds. G. W. Gray, J. W. Goodby and A. Fukuda, Taylor& Francis, London, 1997

[cit37] Chandrasekhar S. , Liquid Crystals, Cambridge Univ. Press, NY, 1992

[cit38] Paraschiv I., Giesbers M., van Lagen B., Grozema F. C., Abellon R. D., Siebbeles L. D. A., Marcelis A. T. M., Zuilhof H., Sudhölter E. J. R. (2006). Chem. Mater..

[cit39] Kao K. C. , Dielectric Phenomena in Solids, Elsevier, Amsterdam, 2004

[cit40] Bai Y. F., Zhao K. Q., Hu P., Wang B. Q., Shimizu Y. (2009). Mol. Cryst. Liq. Cryst..

[cit41] Yoshio M., Mukai T., Ohno H., Kato T. (2004). J. Am. Chem. Soc..

[cit42] Kato T. (2002). Science.

[cit43] Iino H., Hanna J., Haarer D. (2005). Phys. Rev. B.

