# Supplementary material for: Tubular and lamellar hydrogen-bonding molecular assemblies of isophthalic acid derivatives bearing a –CONHCnH2n+1 chain
Source: RSC Adv. 2018 Jun 19;8(39):22250–8. doi: 10.1039/c8ra04077j (PMC9081117; doi:10.1039/c8ra04077j)
Supplement: RA-008-C8RA04077J-s001 [file RA-008-C8RA04077J-s001.pdf]

Electronic Supplementary Material (ESI) for RSC Advances.
This journal is © The Royal Society of Chemistry 2018

Supporting Information

Tubular and Lamellar Hydrogen-Bonding Molecular Assemblies of Isophthalic Acid Derivatives Bearing a –CONHC$_n$H$_{2n+1}$ Chain

Chao Lv, Takashi Takeda, Norihisa Hoshino, and Tomoyuki Akutagawa

Contents

1. Experimental section
2. OG states of **C10IP**, **C14IP** and **C18IP** and their thermodynamic stability/physical strength.
3. SEM images of CS and XG states of **C14IP** on silicon.
4. Variation of the weight-losses for XG states of (**C14IP**)$_6$•(H_2O)$_n$ and (**C18IP**)$_6$•(H_2O)$_n$ based on the TG diagrams.
5. TG and DSC charts of **C6IP** and **C10IP**.
6. XRD patterns of **C6IP**, **C10IP**, **C14IP**, and **C18IP** at room temperature.
7. Vibrational spectra of **C6IP** and **C10IP** on KBr pellets.
8. Vibrational spectra of unhydrated **C14IP** and (**C14IP**)$_6$•(H_2O)$_n$.
9. Vibrational spectra of unhydrated **C18IP** and (**C18IP**)$_6$•(H_2O)$_n$.
10. Elemental analyses and the formula of (**C14IP**)$_6$•(MX) derivatives.

1. Experimental section

Preparation of C10IP. The mixture of benzene-1,3,5-benzenetricarboxylic acid dimethyl ester (2.17 g, 9.11 mmol) in $SOCl_2$ (80 mL, 1.10 mmol) was refluxed for 20 h. After the mixture was cooled to room temperature, excess $SOCl_2$ was removed by evaporation under reduced pressure. The obtained acid chloride was dissolved in dry CH_2Cl_2 (40 mL). Decylamine (1.83 mL, 9.24 mmol), followed by triethylamine (1.28 mL, 9.24 mmol) were added dropwise in 10 min. The mixture was stirred at room temperature for 18 h. The resulting solution was diluted with CH_2Cl_2 (80 mL), and washed with aqueous 1M HCl (110 ml × 3) and brine, and then dried over Na_2SO_4. Evaporation of the solvent under reduced pressure gave the white solid, which was purified by gel permeation chromatography with $CHCl_3$ as eluent to yield compound *N*-decyl-3,5-bis(methoxylcarbonyl)benzamide (**C10MIP**) (928 mg, 29%) as a white solid. The product was used for the next reaction without further purification. ^{1}H NMR (400 MHz, $CDCl_3$): δ 0.88 (t, J = 6.8 Hz, 3H, -CH_3), 1.20-1.45 (m, 14H, alkyl), 1.58-1.69 (m, 2H, -NHCH$_2$CH_2), 3.45-3.52 (m, 2H, -NHCH_2CH$_2$), 3.98 (s, 6H, 2Ar-COOCH_3), 6.22 (br s, 1H, N*H*), 8.61 (d, J = 1.6 Hz, 2H, Ar*H*), 8.79 (t, J = 1.6 Hz, 1H, Ar*H*).

A solution of LiOH・H_2O (413 mg, 9.84 mmol) in water (5 mL) was added the suspension of **C10MIP** (928 mg, 2.46 mmol) in MeOH (20 ml). The suspension was heated to 50 ^oC for 2 h to dissolve all compound, then stirred at room temperature for 18 h. The mixture was poured into water (200 ml) and washed with AcOEt to remove unreacted starting material. The aqueous phase was acidified with aqueous 1M HCl so that the pH turned to 1. The acidic aqueous solution was extracted with AcOEt. The extracted organic phase was dried over Na_2SO_4. Evaporation of the solvent under reduced pressure gave the crude product. The crude product is recrystallized from mixed solvent of CH_3OH-H_2O (5:1) to give the compound **C10IP** (760 mg, 63%) as a white solid. M.p. = 245–246 °C. ^{1}H NMR (400 MHz, DMSO-d_6): δ 0.84 (t, J = 6.8 Hz, 3H, -CH_3), 1.16-1.35 (m, 14H, alkyl), 1.48-1.59 (t, J = 6.8 Hz, 2H, -NHCH$_2$CH_2), 3.27 (q, J = 6.5 Hz, 2H, -NHCH_2), 8.56 (t, J = 1.6 Hz, 1H, Ar*H*), 8.63 (t, J = 1.6 Hz, 2H, Ar*H*), 8.87 (t, J = 5.2 Hz, 1H, -N*H*), 13.50 (s, 2H, -(C=O)-O*H*). IR: ν (cm^{-1}): 3300 (N-H stretch), 1720 and 1695 (C=O of COOH, C=O stretching vibration of the free, non-hydrogen bonded and laterally hydrogen-bonded COOH groups, respectively), 1640 (C=O of CONH), 1544 (C-N). Elemental analysis: calculated for $C_{19}H_{27}NO_5$: C, 65.31; H, 7.79; N, 4.01. Found: C, 65.28; H, 7.91; N, 4.00. HRMS (FAB): calculated for $C_{19}H_{28}NO_5$ m/z 350.1967 [$(M+H)^+$], found m/z 350.1972.

Preparation of C14IP. The mixture of benzene-1,3,5-benzenetricarboxylic acid dimethyl ester (1.00 g, 4.20 mmol) in $SOCl_2$ (40 mL) was refluxed for 20 h. After the mixture was cooled to room

temperature, excess $SOCl_2$ was removed by evaporation under reduced pressure. The obtained acid chloride was dissolved in dry CH_2Cl_2 (20 mL). To the solution tetradecylamine (984 mg, 4.61 mmol), followed by triethylamine (630 μL, 4.52 mmol) were added dropwise in 10 min. The mixture was stirred at room temperature for 18 h. The resulting solution was diluted with CH_2Cl_2 (40 mL), and washed with aqueous 1M HCl (110 ml × 3) and brine, and then dried over Na_2SO_4. Evaporation of the solvent under reduced pressure gave the white solid, which was recrystallized from EtOH to yield *N*-tetradecyl-3,5-bis(methoxylcarbonyl)benzamide (**C14MIP**) (1.08 g, 73%) as a white solid. The product was used for the next reaction without further purification. ^{1}H NMR (400 MHz, $CDCl_3$): δ 0.88 (t, J = 6.4 Hz, 3H, C*H₃*), 1.21-1.44 (m, 22H, alkyl), 1.59-1.69 (m, 2H, -NHCH$_2$CH_2), 3.44-3.53 (m, 2H, -NHCH_2CH$_2$), 3.98 (s, 6H, 2Ar-COOCH_3), 6.21 (br s, 1H, N*H*), 8.61 (d, J = 1.6 Hz, 2H, Ar*H*), 8.79 (t, J = 1.6 Hz, 1H, Ar*H*).

A solution of LiOH・H_2O (420 mg, 10.0 mmol) in water (5 mL) was added the suspension of **C14MIP** (1.08 g, 2.49 mmol) in MeOH (20 ml). The suspension was heated to 50 ^{o}C for 2 h to dissolve all compound, then stirred at room temperature for 18 h. The mixture was poured into water (250 ml). An aqueous HCl (1 M) was added until the pH turned 1. The resulting precipitation was collected by filtration, washed with H_2O and dried under vacuum. Recrystallization of the precipitation from AcOEt gave compound **C14IP** (646 mg, 64%) as a white solid. M.p.: decomposed at above 241 °C. ^{1}H NMR (400 MHz, DMSO-d_6): δ 0.85 (t, J = 6.8 Hz, 3H, CH_3), 1.18-1.35 (m, 22H, alkyl), 1.48-1.58 (m, 2H, CONHCH$_2$CH_2), 3.29 (m, 2H, CONHCH_2), 8.56 (t, J = 1.6 Hz, 1H, Ar*H*), 8.62 (s, 2H, Ar*H*), 8.85 (br t, J = 4.8 Hz, 1H, N*H*), 13.47 (br s, 2H, (C=O)-O*H*). IR: ν (cm^{-1}): 3300 (N-H stretch), 1720 and 1695 (C=O of COOH, C=O stretching vibration of the free, non-hydrogen bonded and laterally hydrogen-bonded CO_2H groups, respectively), 1637 (C=O of CONH), 1537 (C-N). Elemental analysis calculated for $C_{23}H_{35}NO_5$: C, 68.12; H, 8.70; N, 3.45, Found: C, 68.37; H, 8.96; N, 3.57. HRMS (FAB): calculated for $C_{23}H_{36}NO_5$ m/z 406.2593 [(M+H)$^+$], found m/z 406.2594.

Preparation of C18IP. The mixture of benzene-1,3,5-benzenetricarboxylic acid dimethyl ester (2.01 g, 8.40 mmol) in $SOCl_2$ (80 mL) was refluxed for 20 h. After the mixture was cooled to room temperature, excess $SOCl_2$ was removed by evaporation under reduced pressure. The obtained acid chloride was dissolved in dry CH_2Cl_2 (40 mL). To the solution octadecylamine (2.93 g, 9.24 mmol), followed by triethylamine (1.28 mL, 9.24 mmol) were added dropwise in 10 min. The mixture was stirred at 50 ^{o}C for 18 h. The resulting suspension was filtered and dried to obtain the crude product. The crude product was purified with a silica gel column chromatography using $CHCl_3$/AcOEt (10:1) as an eluent to give the compound *N*-octadecyl-3,5-bis(methoxylcarbonyl)benzamide (**C18MIP**)

(2.40 g, 58%) as a white solid. The product was used for the next reaction without further purification. ^{1}H NMR (400 MHz, $CDCl_3$): δ 0.85 (t, J = 6.8 Hz, 3H, CH_3), 1.21-1.44 (m, 30H, alkyl), 1.60-1.70 (m, 2H, -NHCH$_2$CH_2), 3.45-3.52 (m, 2H, -NHCH_2CH$_2$), 3.98 (s, 6H, 2Ar-COOCH_3), 6.22 (s, 1H, NH), 8.61 (d, J = 1.6 Hz, 2H, ArH), 8.80 (t, J = 1.6 Hz, 1H, ArH).

A solution of LiOH・H_2O (786 mg, 18.7 mmol) in water (5 mL) was added the suspension of **C18MIP** (2.20 g, 4.49 mmol) in MeOH (40 ml). The suspension was heated to 50 ^{o}C and stirred at this temperature for 18 h. The mixture was poured into water (250 ml). The aqueous solution was extracted by AcOEt to remove unreacted starting material. The aqueous phase was acidified with aqueous 1M HCl so that the pH turned 1. The resulting precipitation was collected by filtration, washed with H_2O and dried under vacuum. Recrystallization of the precipitation from CH_3OH gave compound **C18IP** (1.77 g, 83%) as a white solid. M.p.: decomposed at above 231 °C. ^{1}H NMR (400 MHz, DMSO-d_6): δ 0.85 (t, J = 6.8 Hz, 3H, -CH_3), 1.17-1.35 (m, 30H, alkyl), 1.47-1.58 (m, 2H, -NHCH$_2$CH_2), 3.23-3.29 (m, 2H, -NHCH_2), 8.56 (t, J = 1.6 Hz, 1H, ArH), 8.63 (s, 2H, ArH), 8.87 (t, J = 5.5 Hz, 1H, -NH), 13.47 (br s, 2H, -(C=O)-OH). IR: ν (cm^{-1}): 3303 (N-H stretch), 1720 and 1695 (C=O of COOH, C=O stretching vibration of the free, non-hydrogen bonded and laterally hydrogen-bonded CO_2H groups, respectively), 1637 (C=O of CONH), 1537 (C-N). Elemental analysis: calculated for $C_{27}H_{43}NO_5$: C, 70.25; H, 9.39; N, 3.03. Found: C, 69.97; H, 9.45; N, 3.15. HRMS (FAB): calculated for $C_{27}H_{44}NO_5$ m/z 462.3219 [$(M+H)^+$], found m/z 462.3219.

2. OG states of C10IP, C14IP and C18IP and their thermal stability and physical strength.

Thermodynamic stability and physical strength of OG states of **C10IP**, **C14IP**, and **C18IP** were evaluated. Figure S1 shows each OG state with horizontally inversed state. The OG state of **C10IP** was stable at 0 °C, which could not maintain enough time at room temperature (Figure S1a). Different from **C10IP**, the OG states of **C14IP** and **C18IP** bearing much longer alkyl chains could form stable organogels at room temperature, and there was a slight difference for the OG states between **C18IP** and **C14IP** (Figure S1). Horizontally inversed vial of OG of **C18IP** did not show a deformation, whereas that of **C14IP** indicated a slight deformation due to the gravity. Therefore, the strength of oraganogels was increased in the order of **C10IP**, **C14IP**, to **C18IP**, which was clearly correlated with the alkyl chain length and also hydrophobic interaction.

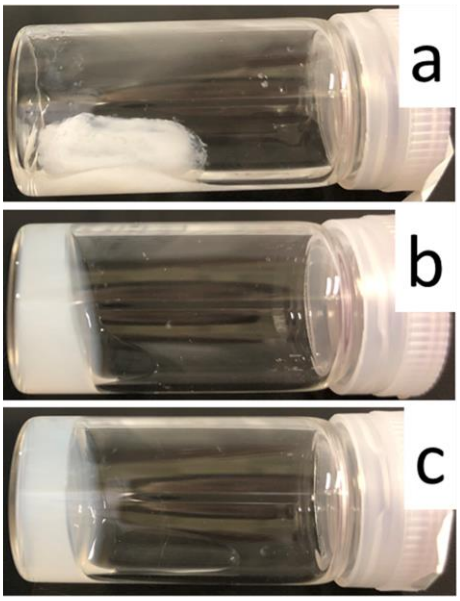

Figure S1. OG states of a) **C10IP**, b) **C14IP** and c) **C18IP** at room temperature with a fixed concentration of 10 mM and minimum H_2O volume percentages of 50, 40, and 30 %, respectively.

3. SEM images of CS and XG states of C14IP on silicon

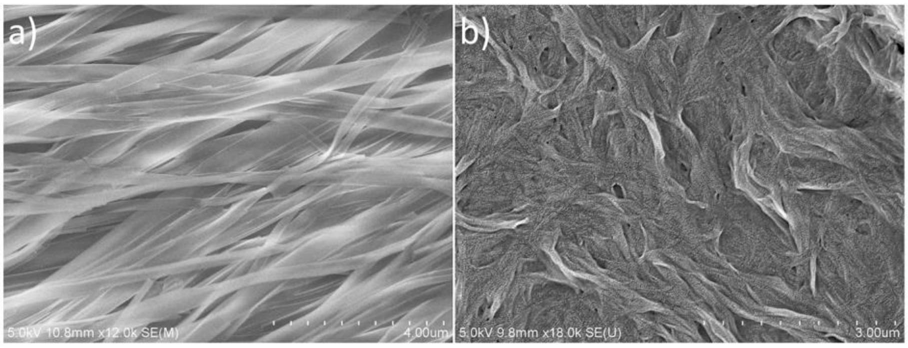

Figure S2. SEM images of CS and XG states of **C14IP** on silicon. a) CS state of unhydrated **C14IP**, and b) XG state of **C14IP** on silicon.

4. Variation of the weight-losses for XG states of (C14IP)$_6$•(H_2O)$_n$ and (C18IP)$_6$•(H_2O)$_n$ based on the TG diagrams

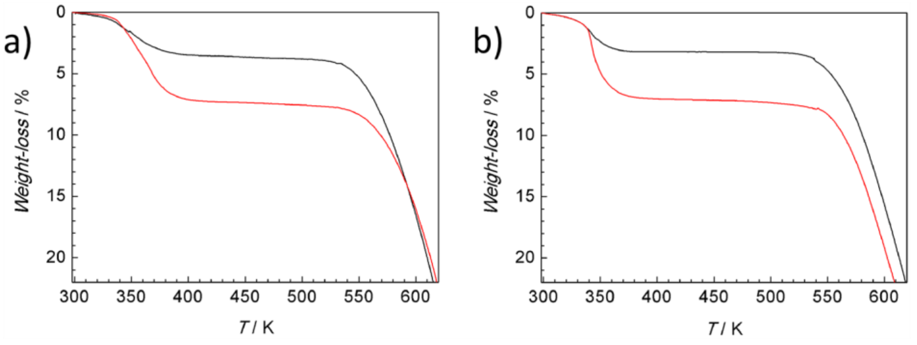

Figure S3. Variation of the weight-losses for XG states of a) (**C14IP**)$_6$•(H_2O)$_n$ and b) (**C18IP**)$_6$•(H_2O)$_n$ based on the TG diagrams.

5. TG and DSC charts of C6IP and C10IP

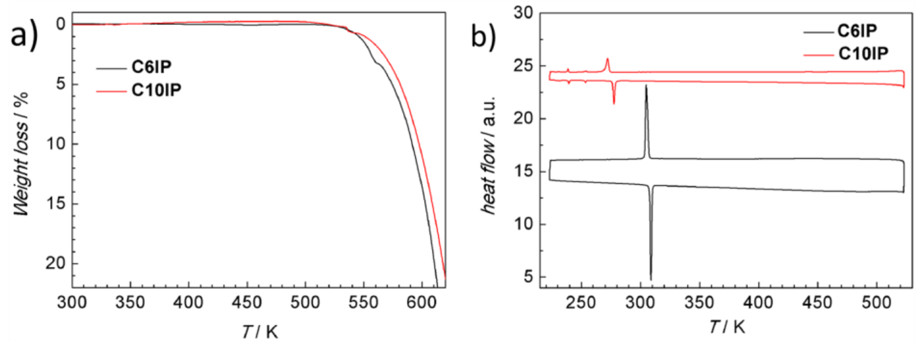

Figure S4. TG and DSC charts of **C6IP** and **C10IP**. a) TG of **C6IP** and **C10IP**, b) DSC of **C6IP** and **C10IP**.

6. XRD patterns of C6IP, C10IP, C14IP, and C18IP at room temperature

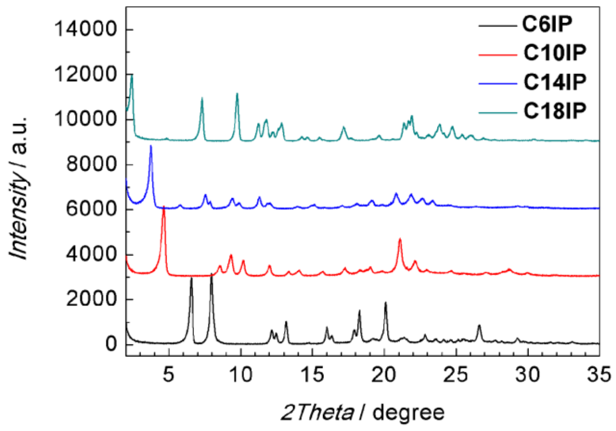

Figure S5. XRD patterns of **C6IP**, **C10IP**, **C14IP**, and **C18IP** at room temperature.

7. Vibrational spectra of C6IP and C10IP on KBr pellets.

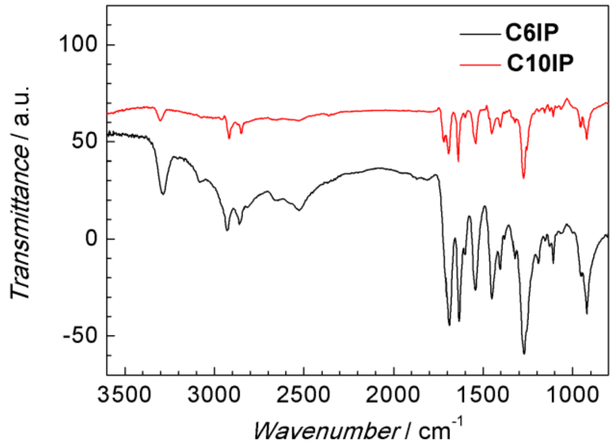

Figures S6. Vibrational spectra of **C6IP** and **C10IP** on KBr pellets.

8. Vibrational spectra of unhydrated C14IP and $(C14IP)_6•(H_2O)_n$.

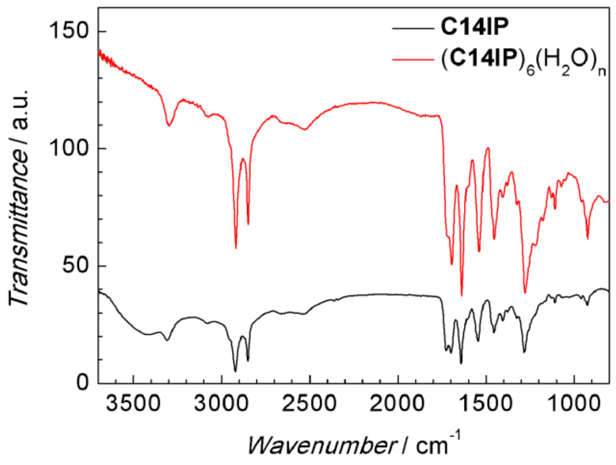

Figure S7. Vibrational spectra of unhydrated **C14IP** and (**C14IP**)$_6$•$(H_2O)_n$.

9. Vibrational spectra of unhydrated C18IP and $(C18IP)_6•(H2O)_n$.

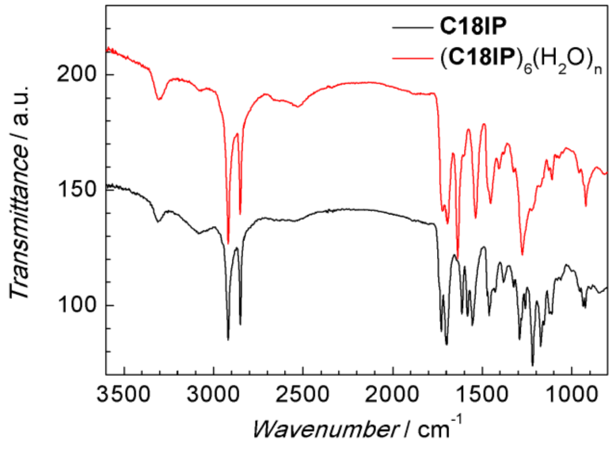

Figure S8. Vibrational spectra of unhydrated **C18IP** and (**C18IP**)$_6$•$(H_2O)_n$.

10. Elemental analyses and the formula of (C14IP)$_6$•(MX) derivatives.

Table S1. Elemental analyses and the formula of (**C14IP**)$_6$•(LiCl), (**C14IP**)$_6$•(NaCl-H_2O), (**C14IP**)$_6$•(KCl-4H_2O), and (**C14IP**)$_6$•(H_2SO_4-3H_2O).

Formula	calcd.	found
(**C14IP**)$_6$•(LiCl)	C 66.95, H 8.55, N 3.39	C 66.89, H 8.73, N 3.64
(**C14IP**)$_6$•(NaCl-H_2O)	C 66.05, H 8.51, N 3.35	C 66.07, H 8.75, N 3.56
(**C14IP**)$_6$•(KCl-4H_2O)	C 65.88, H 8.69, N 3.29	C 65.84, H 8.69, N 3.42
(**C14IP**)$_6$•(H_2SO_4-3H_2O)	C 64.11, H 8.50, N 3.25	C 63.89, H 8.56, N 3.34
